# Molecular characterisation and the protective immunity evaluation of *Eimeria maxima* surface antigen gene

**DOI:** 10.1186/s13071-018-2906-5

**Published:** 2018-05-30

**Authors:** Tingqi Liu, Jingwei Huang, Yanlin Li, Muhammad Ehsan, Shuai Wang, Zhouyang Zhou, Xiaokai Song, Ruofeng Yan, Lixin Xu, Xiangrui Li

**Affiliations:** College of Veterinary Medicine, Nanjing Agriculture University, 1 Weigang, Nanjing, Jiangsu 210095 People’s Republic of China

**Keywords:** *Eimeria maxima*, Surface antigen, Cytokines, Vaccine, Immunity

## Abstract

**Background:**

Coccidiosis is recognised as a major parasitic disease in chickens. *Eimeria maxima* is considered as a highly immunoprotective species within the *Eimeria* spp. family that infects chickens. In the present research, the surface antigen gene of *E. maxima* (EmSAG) was cloned, and the ability of EmSAG to stimulate protection against *E. maxima* was evaluated.

**Methods:**

Prokaryotic and eukaryotic plasmids expressing EmSAG were constructed. The EmSAG transcription and expression *in vivo* was performed based on the RT-PCR and immunoblot analysis. The expression of EmSAG in sporozoites and merozoites was detected through immunofluorescence analyses. The immune protection was assessed based on challenge experiments. Flow cytometry assays were used to determine the T cell subpopulations. The serum antibody and cytokine levels were evaluated by ELISA.

**Results:**

The open reading frame (ORF) of EmSAG gene contained 645 bp encoding 214 amino acid residues. The immunoblot and RT-PCR analyses indicated that the EmSAG gene were transcribed and expressed *in vivo*. The EmSAG proteins were expressed in sporozoite and merozoite stages of *E. maxima* by the immunofluorescence assay. Challenge experiments showed that both pVAX1-SAG and the recombinant EmSAG (rEmSAG) proteins were successful in alleviating jejunal lesions, decreasing loss of body weight and the oocyst ratio. Additionally, these experiments possessed anticoccidial indices (ACI) of more than 170. Higher percentages of CD4^+^ and CD8^+^ T cells were detected in both EmSAG-inoculated birds than those of the negative control groups (*P* < 0.05). The EmSAG-specific antibody concentrations of both the rEmSAG and pVAX1-EmSAG groups were much higher than those of the negative controls (*P* < 0.05). Higher concentrations of IL-4, IFN-γ, TGF-β1 and IL-17 were observed more in both the rEmSAG protein and pVAX1-SAG inoculated groups than those of negative controls (*P* < 0.05).

**Conclusions:**

Our findings suggest that EmSAG is capable of eliciting a moderate immune protection and could be used as an effective vaccine candidate against *E. maxima*.

## Background

Coccidiosis is recognised as a major parasitic disease in chickens seriously affecting the efficiency of feed conversion and leading to decreased production. *Eimeria maxima* has been recognised as one of the most economically significant species of *Eimeria* [1]. Currently, prophylactic chemotherapy with anticoccidial drugs is the major control strategy for coccidiosis. Traditional anticoccidial drugs and live vaccines have their own defect [2]. Subunit vaccines encoding the *Eimeria* proteins which stimulated protective immunity were accepted as effective vaccines against coccidiosis [3–5]. Recently, many reports have shown that cell-mediated immunity could be stimulated by DNA vaccines [6–10].

Surface antigens have been proven to confer protection against coccidiosis by altering key processes in host cell invasions [11]. The SAGs protein of *Eimeria tenella* is capable of inducing an immune response against coccidiosis in chickens [12]. Therefore, surface antigens and cell adhesion proteins have been suggested as promising vaccine candidates against parasitic infections [13, 14].

*Eimeria maxima* is regarded as a highly immunoprotective species within the family of *Eimeria* spp. affecting chickens [15–19]. In this study, subunit and DNA vaccines made from EmSAG were evaluated for their protection against *E. maxima*.

## Methods

### Chickens and parasites

*Eimeria*-free birds at one day of age were reared in captivity with provided water and feed *ad libitum*. The birds were placed in a coccidia-free environment. The Jiangsu strain of *E. maxima* was developed and maintained in *Eimeria*-free birds our laboratory. Sporozoites from *E. maxima* oocysts were cleaned and sporulated as previously described [20].

### Amplification and prokaryotic expression of EmSAG

The construction of prokaryotic expression of EmSAG was conducted as previously described [20]. Briefly, the EmSAG-encoding sequence (GenBank: XM_013482011.1) was amplified by PCR. EmSAG-specific primers were utilised for the PCR assays: SAG1 (forward primer: 5'-CGC GGA TCC GAC ACA ATC TCC AGC CCT-3'; *BamH*I restriction sites underlined) and SAG2 (reverse primer: 5'-ATT GCG GCC GCT CAA ATG AGA ACA GAT GCG-3'; *Not*I restriction sites underlined) with *E. maxima* cDNA as a template. The amplification products of EmSAG cloned in pMD-19T (TaKaRa, Dalian, China) resulted in the formation of recombinant plasmid pMD-19T-EmSAG. Subsequently, the EmSAG gene was inserted into the pET-32a (+) (Novagen, Madison, WI, USA) frame of expression vector system and confirmed by endonuclease digestion. Following sequence analysis (Invitrogen Biotech, Shanghai, China) and verification, the positive clones were confirmed as pET-32a/ EmSAG.

### Expression of the recombinant EmSAG protein

The sequence identity of EmSAG was compared to the known SAG sequences of other *Eimeria* spp. and assessed using the BLASTx and BLASTp search tools (http://blast.ncbi.nlm.nih.gov/Blast.cgi). The amino acid sequence of EmSAG was used to predict N-terminal signal peptides through a bioinformatics online program (http://www.cbs.dtu.dk/services/SignalP/). The cladogram was made using the MEGA 6.0 programme with the neighbour-joining method. The pET-32a/EmSAG was expressed in *E. coli* BL21 (DE3) as described previously [21]. The recombinant protein was purified and the concentration of the sample was determined using the Bradford method [22]. The rEmSAG protein was kept frozen (-70 °C) until further analysis.

### Development of anti-EmSAG antibodies against the rEmSAG protein

Rat polyclonal anti-EmSAG antibodies were generated in the Sprague-Dawley rats at 4 weeks of age. Rats were subcutaneously immunised with a total of 0.3 mg of rEmSAG protein mixed with Freund’s complete adjuvant. Fourteen days after the first immunisation, the rats were given a booster injection with 0.3 mg of rEmSAG protein in Freund’s incomplete adjuvant. Three booster doses were given at 1-week intervals. Finally, rat serum containing antibodies were obtained after the last booster injection and kept frozen (at -70 °C) until subsequent analysis. Pre-immunisation serum was obtained for later use as the negative control [23].

### Construction of eukaryotic plasmid of EmSAG

The construction of eukaryotic plasmid of EmSAG was conducted and purified as previously described [20]. Briefly, the EmSAG fragment was inserted into the pVAX1, following the sequence analysis (Invitrogen Biotech) and verification, the positive clones were confirmed as pVAX1-EmSAG. The plasmids encoding EmSAG were extracted using EndoFree Plasmid MEGA Kit (Qiagen, Valencia, CA, USA). The concentration of the sample was determined using as per the method suggested earlier [20]. Finally, the plasmids were kept frozen (-20 °C) until subsequent analysis.

### Immunoblot analysis of native EmSAG and rEmSAG proteins

Immunoblot analyses for rEmSAG and native EmSAG were performed as described in an earlier work [20]. Rat anti-rEmSAG sera (dilutions of 1:200) were used to detect sporozoites. Chicken anti-*E. maxima* sera (dilutions of 1:100) were used to detect the rEmSAG protein. Goat anti-rat HRP-IgG and donkey anti-chick HRP-IgG (Sigma-Aldrich, St. Louis, MO, USA) were used as a secondary antibody.

### Transcription and expression of pVAX1-EmSAG *in vivo*

The EmSAG transcription *in vivo* was performed based on the RT-PCR and immunoblot analysis, as previously described [24]. Briefly, in coccidia-free chickens, a total of 100 μg pVAX1-EmSAG plasmid was intramuscularly injected into the legs. In the pVAX1 control group, the 100 μg pVAX1 plasmid was injected into the legs. One week post-immunisation, the tissues from vaccinated and non-vaccinated chickens were collected for both RT-PCR and immunoblot analyses. EmSAG-specific primers were utilised for the RT-PCR assays. Rat anti-rEmSAG sera (dilutions of 1:200) were used to detect pVAX1-EmSAG expression. The secondary antibody was HRP-conjugated goat anti-rat IgG (Sigma-Aldrich).

### Location of the EmSAG protein in sporozoites and merozoites stages

Immunofluorescence technique was used to locate EmSAG in sporozoites and merozoites as previously described [25]. Nuclei were probed with 2-(4-amidinophenyl)-6-indole carbamidinedihydrochloride (DAPI, Sigma-Aldrich). Rat anti-EmSAG sera (1:100 dilutions) were used as the primary antibody. The secondary antibody was Cy3-conjugated goat anti-rat IgG (Beyotime, Haimen, Jiangsu, China) (dilution of 1:1000). The slides were analysed using fluorescence microscopy (Nikon, Tokyo, Japan).

### Experimental design

Chickens at 14 days of age, negative for *Eimeria* were placed in six groups, each including 30 birds. The chickens were inoculated intramuscularly injection with the pVAX1-SAG (100 μg/chick) or rEmSAG protein (200 μg/chick). In the pVAX1 control chickens, a total of 100 μg pVAX1 plasmid was injected into the legs. In the pET-32a control chickens group, a total of 200 μg pET-32a protein was injected as above. The challenged and unchallenged control birds were immunised with PBS. One week later, the birds were boosted with the same route as the primary immunisation. Subsequently, 7 days after the last immunisation, 1 × 10^5^ sporulated oocysts of *E. maxima* were given to all the birds except the negative control birds. Seven days later, the birds were euthanised to measure their immune response and degree of coccidial protection. Moreover, the birds (*n* = 5 per group) were placed in another coccidia-free room. Finally, 10 days after the last immunisation, the serum samples were harvested and kept frozen (-20 °C) until further antibodies and cytokine production analysis could be conducted.

### Assessment of immune protection

The chickens were monitored for body weight gain and signs of immune protection (jejunal lesion score, survival rate and change in oocyst ratio). Lesion scrapings were microscopically examined for any coccidia, whenever there was doubt of truly coccidia-induced lesions. The jejunal lesion scores of the birds were also evaluated, as described in previous research [23]. The body weight gains were measured at different time-points: the days of vaccination, at the time of the coccidia challenge, and at the end of the test. All the jejunal contents from each bird were harvested and used to evaluate the oocyst counts as described in a previous study [26]. Using the McMaster’s counting method, oocysts and the oocyst ratio were assessed as previously described [27]. Anticoccidial index (ACI) values were evaluated as per the standard formula for assessing protection against *E. maxima* [20].

### ELISA analysis of the serum antibody and cytokine

EmSAG-specific IgY/IgG antibodies were detected by ELISA using the rEmSAG protein as a coating antigen, following previous protocols [28]. The serum samples (1:50 dilution) were detected using the secondary antibodies of donkey anti-chicken HRP-conjugated IgG monoclonal antibody (Sigma-Aldrich). The experiment was completed in duplicate.

For cytokines level analysis, serum samples were obtained and measured as previously described [29]. Briefly, 10 days after the last inoculation, the serum samples of the birds (*n* = 5) per group were harvested to evaluate the cytokines. The titers of IL-4, IL-17, IFN-γ and TGF-β1 were measured using ELISA kits (CUSABIO, Wuhan, China). The data was pooled from three independent experiments.

### Determination of T-cell response

The counts of T cells in the treatment groups were evaluated by flow cytometry analysis as previously described [30, 31]. Spleens were extracted from 5 chickens of each group at pre-, first-, and second-vaccination days. Lymphocytes were obtained from the spleens were stained with SPRD-conjugated CD3 monoclonal antibodies. The cells were then probed with PE-conjugated mouse monoclonal anti-chicken CD4 or the PE-conjugated mouse monoclonal anti-chicken CD8 (Southern Biotechnology Associates, Birmingham, AL, USA). Using FACS flow cytometer, the stained cells were analysed with Cell Quest software (BD Biosciences, San Jose, CA, USA).

### Statistical analysis

All data was expressed as the mean ± standard deviation using the SPSS Statistical Software (SPSS Inc., Chicago, IL, USA). The data were analysed with one-way ANOVA using Duncan’s *post-hoc* test and considered to be statistically significant at *P* < 0.05.

## Results

### EmSAG sequence analysis

**Fig. 1:**
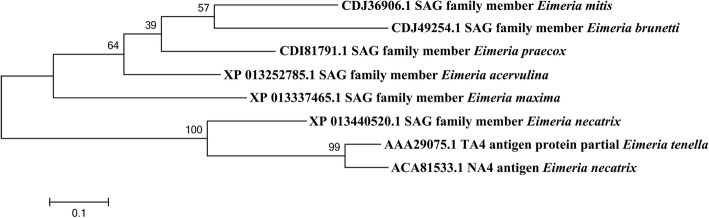
The phylogenetic tree was constructed using CLUSTAL W alignment and neighbour-joining method of the software MEGA 6.0

Using *E. maxima* cDNA as a template, the PCR product of EmSAG was isolated and ligated with pMD19-T. Sequence analysis showed that the EmSAG ORF encoded a protein of 24.73 kDa with a pI of 4.808. As shown in Fig. 1, the phylogenetic tree formulation indicated that the kinship of EmSAG protein was highly related to the EtTA4 and EnNA4 when compared with other *Eimeria* spp. (*E. mitis*, *E. brunetti*, *E. praecox*, *E. acervulina* and *E. necatrix*). The amino acid sequence was analysed with the SignalP programme. The findings suggested an obvious signal peptide possessed a cleavage site between position 21 and 22.

### Purification of the rEmSAG protein

**Fig. 2:**
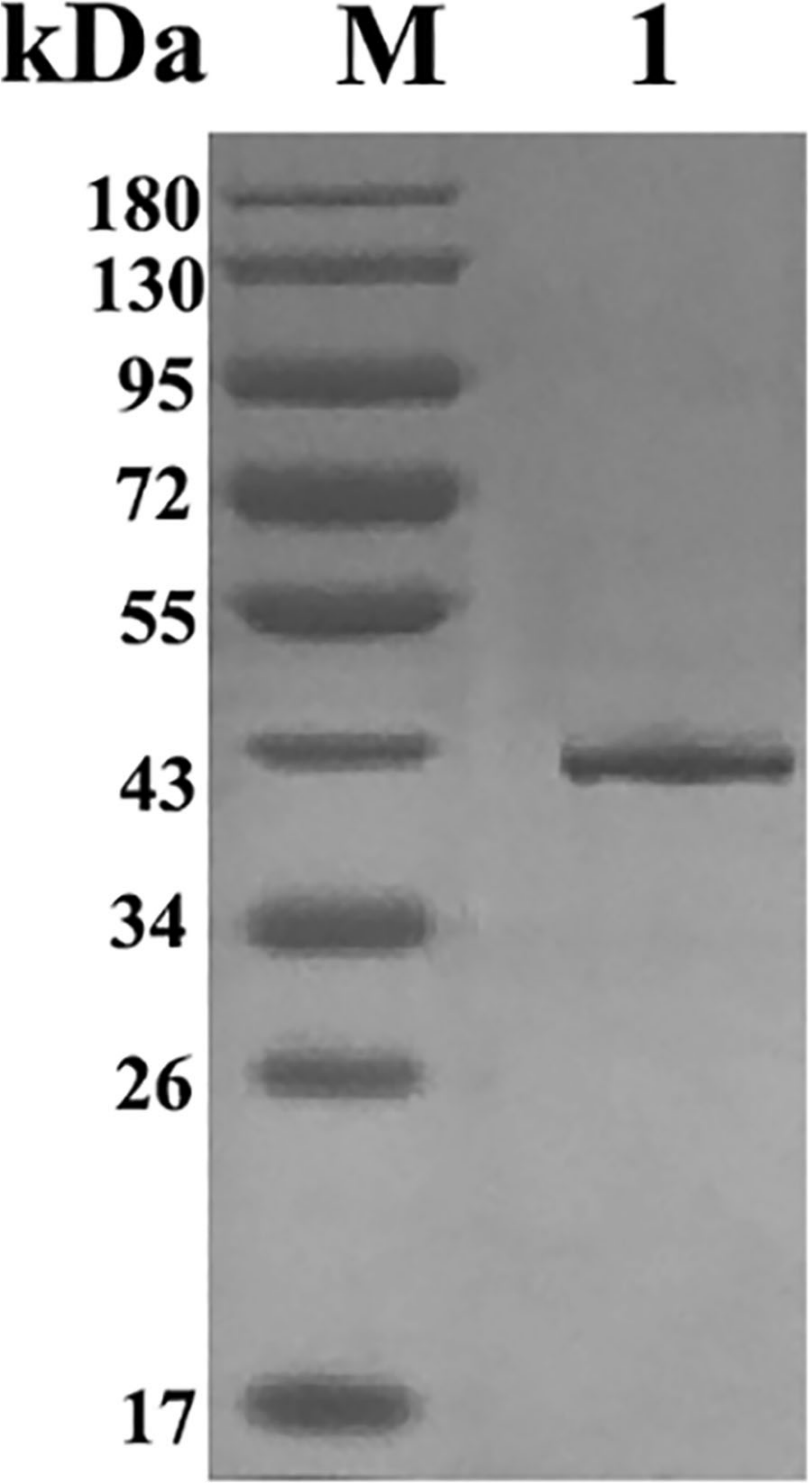
Purified rEmSAG protein separated by SDS-PAGE. Lane M: pre-stained protein marker; Lane 1: the purified rEmSAG protein stained with Coomassie brilliant blue

The pET32a/EmSAG plasmids were expressed in *E. coli* BL21. After IPTG induction, the rEmSAG proteins were harvested. The purified fusion rEmSAG protein was approximately 43 kDa (Fig. 2). This calculated total value of 43 kDa was considered accurate as the sum of both the approximate 20 kDa length of pET-32a (+) and the approximate 23 kDa length of the EmSAG protein.

### Immunoblot analysis of native and rEmSAG proteins

**Fig. 3:**
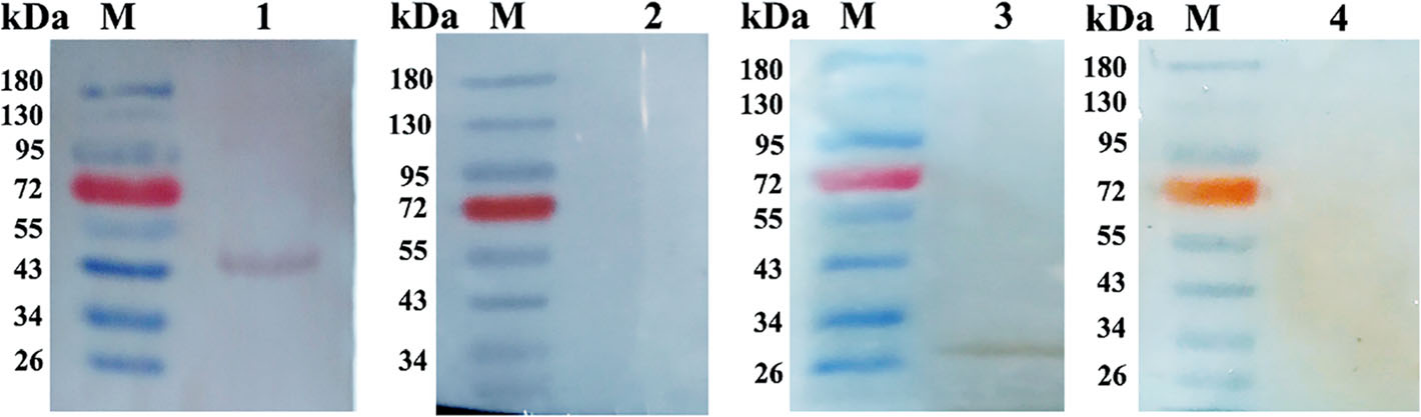
Immunoblot analysis for native and rEmSAG proteins. Lane M: pre-stained protein marker; Lane 1: rEmSAG recognised by chick anti-*E. maxima* serum; Lane 2: rEmSAG protein tested against unimmunised chicken sera; Lane 3: *E. maxima* protein from sporozoites detected by rat anti-rEmSAG sera; Lane 4: protein of *E. maxima* sporozoites detected by unimmunised rat sera

The native and rEmSAG proteins were evaluated by the western blot method (Fig. 3). The rEmSAG protein was tested using chicken *E. maxima*-specific antibodies, but not by the antibodies of unimmunised chickens. Furthermore, the western blot assay also showed a band of almost 26 kDa belonging to the sporozoites protein detected by rat anti-rEmSAG antibodies (Fig. 3), in contrast to the serum from the negative control rats that did not display any bands.

### Identification of EmSAG location in sporozoites and merozoites

**Fig. 4:**
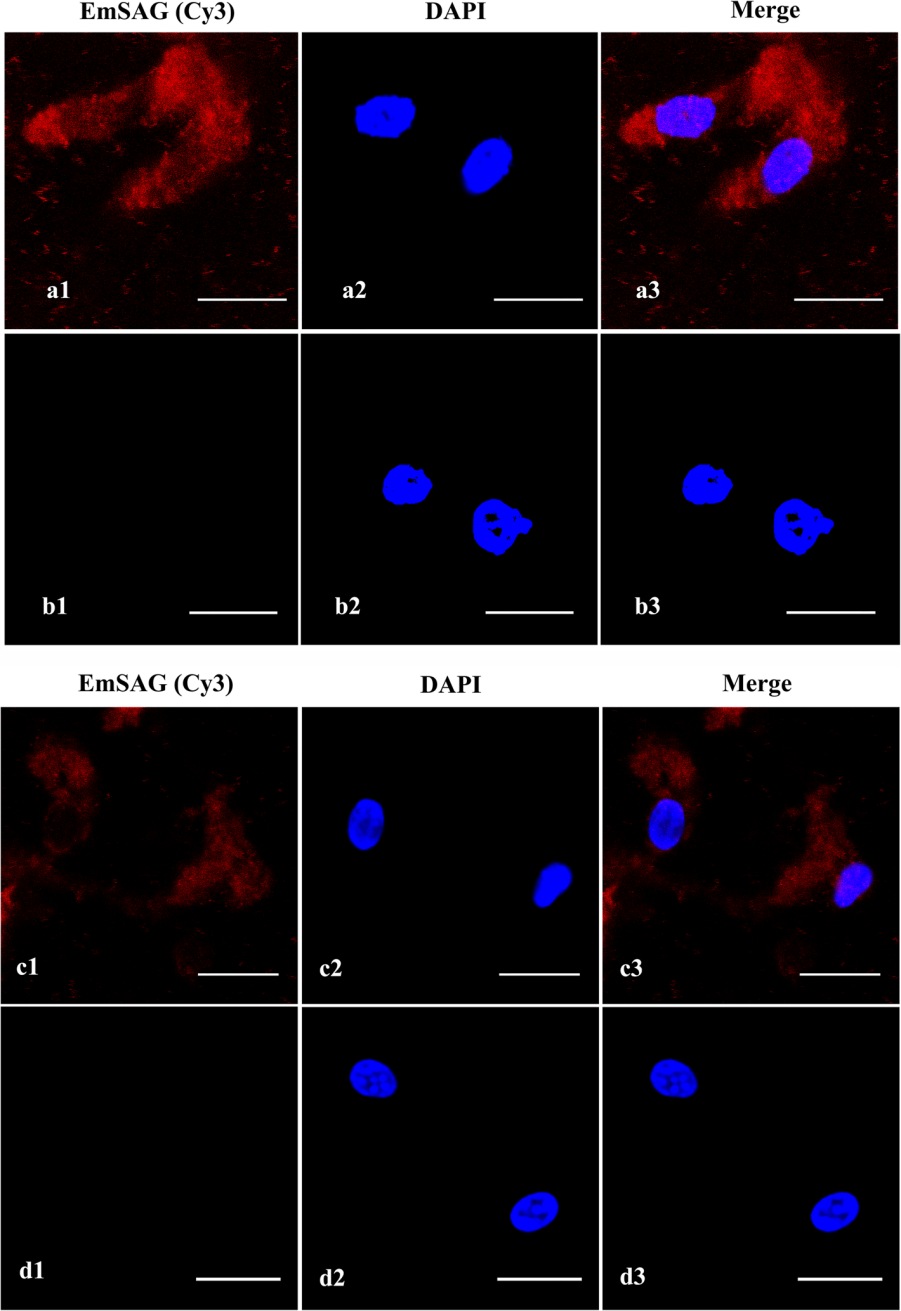
Expression of EmSAG protein in sporozoites and merozoites at 100× magnification. **a** The sporozoites were detected by rat anti-rEmSAG antibodies. **a1** Sporozoites were dyed by Cy3. **a2** The nuclei were probed by DAPI. **a3** Overlaps of Cy3 and DAPI. **b** The sporozoites were detected by unimmunised rat antibodies. **b1** Cy3 stains. **b2** DAPI stains. **b3** Merge. **c** Merozoites were detected by rat anti-rEmSAG antibodies. **c1** Cy3 stains. **c2** DAPI stains. **c3** Merge. **d** The merozoites were detected by unimmunised rat antibodies. **d1** Cy3 stains. **d2** DAPI stains. **d3** Merge. *Scale-bars*: 10 μm

The location of EmSAG in sporozoites and merozoites of *E. maxima* was confirmed using immunofluorescence analyses (Fig. 4). The EmSAG protein was detected using rat anti-rEmSAG antibodies, and Cy3-conjugated goat anti-rat IgG as secondary antibodies shown in red fluorescence, whereas no red fluorescence was detected in cells probed with the pre-immune rat serum. The nuclei of the sporozoites and merozoites were visualised as blue. These results suggest that EmSAG was expressed in both the sporozoite and merozoite stages of *E. maxima.*

### Identification of transcription and expression of pVAX1-EmSAG *in vivo*

**Fig. 5:**
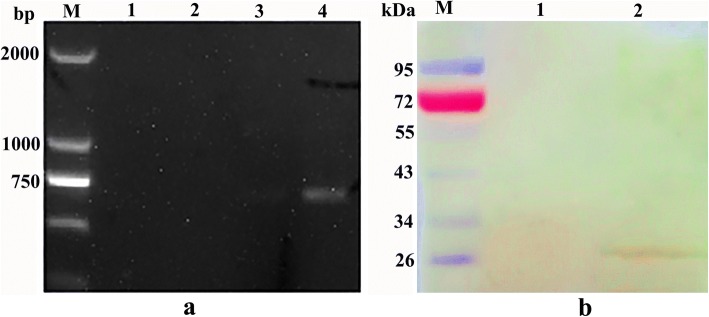
Expression and transcription of pVAX1-SAG *in vivo* were identified through RT-PCR and western blot assays. **a** RT-PCR of pVAX1-EmSAG transcription in chicken muscle. Lane M: DNA marker DL2000; Lanes 1 and 2: the muscle RNA sample from the non-inoculated chicken; Lane 3: the muscle RNA sample from the pVAX1-inoculated chicken; Lane 4: the muscle RNA sample from the pVAX1-EmSAG injected chicken. **b** Western blot of pVAX1-EmSAG in chicken muscle. Lane M: pre-stained protein marker; Lane 1: the protein sample from pVAX1-inoculated chickens; Lane 2: the protein sample from pVAX1- EmSAG inoculated chickens

Transcription and expression of pVAX1-SAG *in vivo* was evaluated through RT-PCR, using the EmSAG-specific primers. A specific DNA band was detected belonging to pVAX1-EmSAG in the tissues of the injected site (Fig. 5a, Lane 4). The RNA samples from non-inoculated and pVAX1-inoculated tissues did not detect any band in the RT-PCR analyses (Fig. 5a, Lanes 1, 2 and 3).

In addition, expression of pVAX1-SAG *in vivo* was detected through immunoblot analysis. A unique band of approximately 26 kDa was detected in the pVAX1-EmSAG-vaccinated muscle sample. In contrast, no band was shown in the pVAX1-immunised muscle samples (Fig. 5b). These results indicate the successful transcription and expression of the EmSAG gene *in vivo*.

### Determination of IgY/IgG and cytokines levels using ELISA

**Fig. 6:**
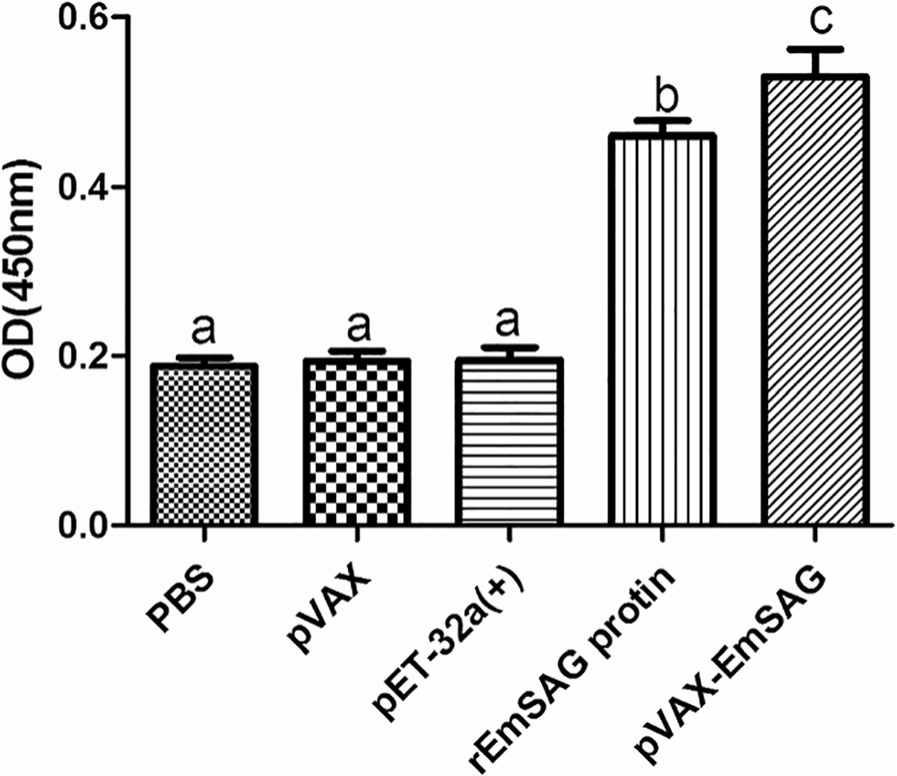
Levels of EmSAG-specific IgY/IgG in chicken sera were measured using ELISA. The titers of the EmSAG-specific IgY/IgG are expressed as the mean + SD

To evaluate the titers of IgY/IgG and the cytokines, serum samples from the immunised birds (*n* = 5 per group) were harvested at 10 days after the last vaccination. The anti-EmSAG IgY/IgG titers of each group are shown in Fig. 6. The IgY/IgG titers of both EmSAG-immunised groups were much higher (ANOVA, *F*_(4, 20)_ = 77.78, *P* < 0.0001) compared to the controls.

**Fig. 7:**
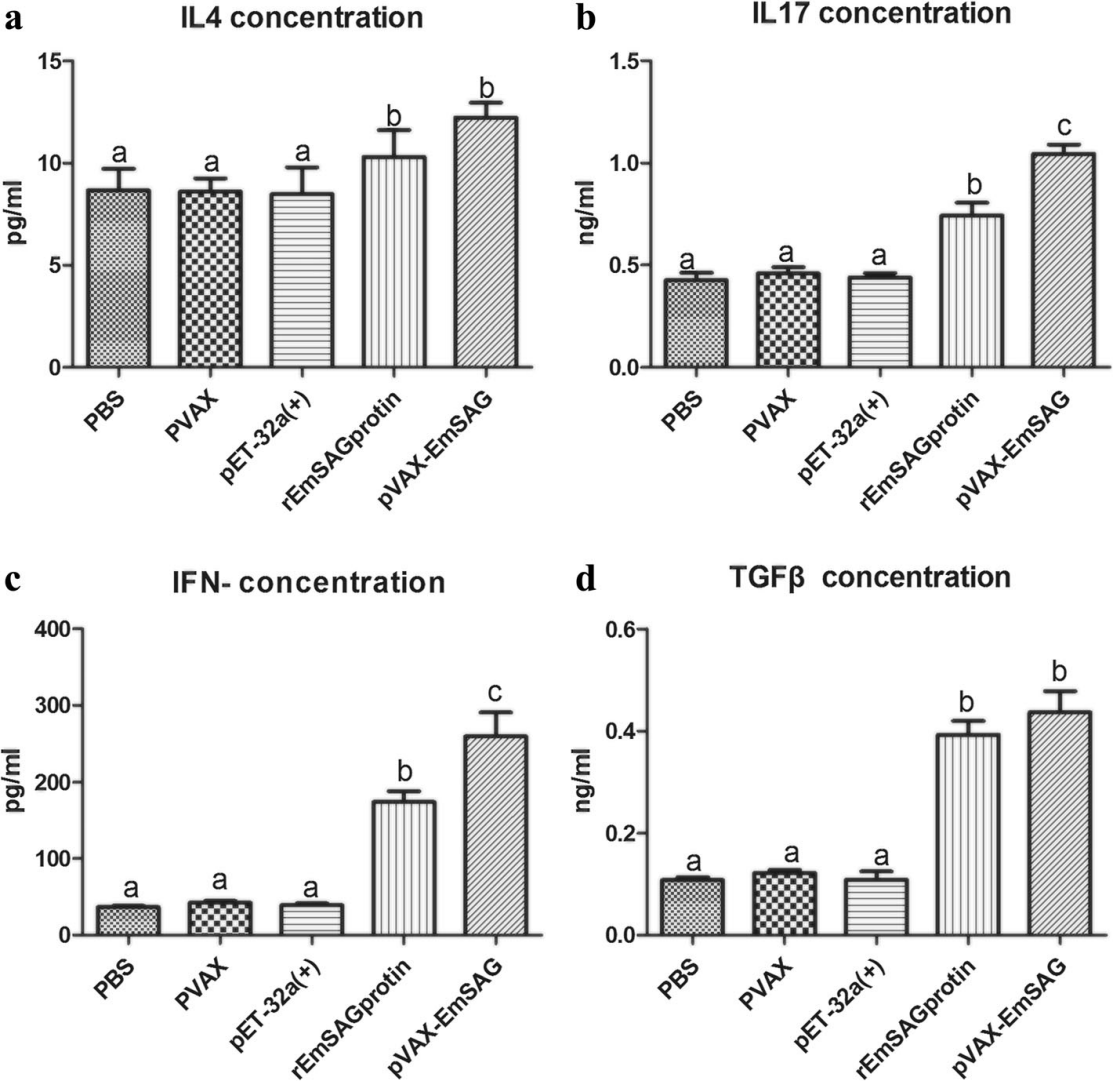
Levels of cytokines IL-4 (**a**), IL-17 (**b**), IFN-γ (**c**) and TGF-β (**d**) in chicken sera were measured using ELISA. Bars with different letters are significantly different (*P* < 0.05)

The titers of cytokines were measured using ELISA (Fig. 7). The serum samples in both pVAX1-EmSAG and rEmSAG-immunised chickens displayed higher titers of IFN-γ (ANOVA, *F*_(4, 20)_ = 43.59, *P* < 0.0001), IL-17 (ANOVA, *F*_(4, 20)_ = 42.25, *P* < 0.0001), IL-4 (ANOVA, *F*_(4, 20)_ = 3.25, *P* = 0.033) and TGF-β1 (ANOVA, *F*_(4, 20)_ = 48.12, *P* < 0.0001) compared to the negative controls.

### Analysis of T cell subpopulations

**Fig. 8:**
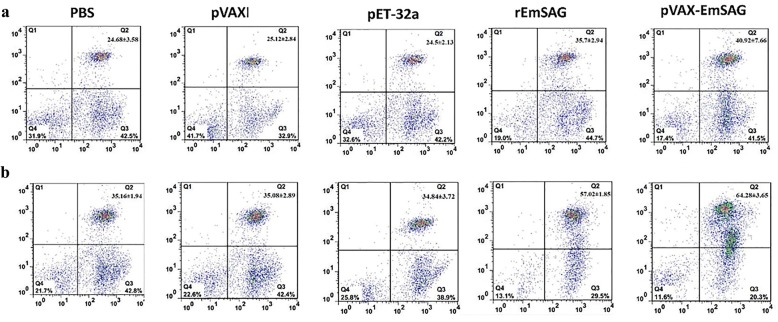
T lymphocytes subpopulations were detected by the flow cytometry technique. **a** CD4^+^ T lymphocytes (CD3^+^CD4^+^, region Q2). **b** CD8^+^ T lymphocytes (CD3^+^CD8^+^, region Q2)

To evaluate the EmSAG specific T-cell responses, flow cytometry assays were used to analyse the CD4^+^ and CD8^+^ T cells. The spleen lymphocytes were collected at pre-, first-, and second-inoculation time-points (Fig. 8). After the last vaccination, the percentage of CD4^+^ in the EmSAG-immunised chickens was higher (ANOVA, *F*_(4, 20)_ = 46.28, *P* < 0.0001), than those in the PBS group, pVAX1.0 group, and the pET-32a (+) group. Regarding CD8^+^ T cells, EmSAG groups showed a higher (ANOVA, *F*_(4, 20)_ = 43.59, *P* < 0.0001) percentage, whereas the PBS, pVAX1 and pET-32a (+) control group remained at low levels after the second immunisation (Table 1 and Fig. 8).

**Table 1:** Flow cytometry analysis of the percentages of T lymphocyte subsets (mean ± SD, %)

Marker	Group	Pre-immunized (%)	1st immunized (%)	2nd immunized (%)
CD4^+^	PBS	23.74 ± 3.21^a^	24.18 ± 1.94^a^	24.68 ± 3.58^a^
	pVAX1.0 control	24.28 ± 3.67^a^	25.26 ± 3.55^a^	25.12 ± 2.84^a^
	pET-32a(+) control	24.04 ± 3.39^a^	23.32 ± 2.88^a^	24.50 ± 2.13^a^
	rEmSAG	23.12 ± 3.4^a^	28.14 ± 4.67^a^	35.70 ± 2.94^b^
	pVAX-EmSAG	24.96 ± 1.66^a^	35.24 ± 3.51^b^	40.92 ± 7.66^b^
CD8^+^	PBS	34.74 ± 3.13^a^	34.18 ± 3.94^a^	35.16 ± 1.94^a^
	pVAX1.0 control	34.64 ± 1.35^a^	35.26 ± 3.65^a^	35.08 ± 2.89^a^
	pET-32a(+) control	35.32 ± 2.57^a^	34.20 ± 5.37^a^	34.84 ± 3.72^a^
	rEmSAG	34.8 ± 6.19^a^	45.44 ± 3.83^b^	57.02 ± 1.85^b^
	pVAX-EmSAG	34.28 ± 5.61^a^	49.04 ± 2.76^b^	64.28 ± 3.65^c^

*Note*: In each column, different letters indicate a significant difference (*P* < 0.05) between numbers. There is no significant difference (*P* > 0.05) between numbers with the same letter

### Immune protection of EmSAG against *E. maxima*

To analyse the immune protection of EmSAG against *E. maxima*, the challenge experiments were assessed. The degrees of immune protection conferred by vaccinations of pVAX1-EmSAG and rEmSAG proteins were measured, and the results of ACI are described in Table 2. Birds inoculated with EmSAG exhibited higher weight gains (ANOVA, *F*_(5, 174)_ = 27.67, *P* < 0.0001) and greater decreases in oocyst ratios when compared to all other groups. The ACIs of the EmSAG-immunised chickens were more than 170, providing moderate protective immunity.

**Table 2:** Effects of SAG against *E. maxima* challenge on different parameters

Group	Average body weight gain (g) (mean ± SD)	Mean lesion scores (mean ± SD)	Oocyst decrease ratio (%)	Anti-coccidial index
Unchallenged control	55.75 ± 18.29^a^	0.00 ± 0.00^a^	100^a^	200
Challenged control	23.84 ± 14.52^b^	2.16 ± 0.13^b^	0^b^	81.12
pVAX1 control	25.16 ± 12.86^b^	2.09 ± 0.18^b^	2.76^b^	84.18
pET 32a control	24.93 ± 14.32^b^	2.13 ± 0.14^b^	2.54^b^	83.36
rEmSAG	48.12 ± 16.10^c^	1.22 ± 0.10^c^	75.93^c^	173.07
pVAX1-SAG	49.17 ± 14.82^c^	1.13 ± 0.16^c^	76.64^c^	175.88

*Note:* In each column, different letters indicate a significant difference (*P* < 0.05) between numbers. There is no significant difference (*P* > 0.05) between numbers with the same letter

## Discussion

In this research, both DNA and recombinant protein vaccines encoding EmSAG of *E. maxima* were compared regarding their abilities to induce protection against *E. maxima* infection. These results indicated that inoculation with EmSAG could promote IgG levels in the sera and upregulated the titers of IL-4, IFN-γ, IL-17 and TGF-β1. Furthermore, the data from the animal experiments proved that EmSAG-immunised groups could produce ACIs of more than 170. Taken together, these data demonstrate that EmSAG vaccines could stimulate moderate protection against *E. maxima*.

DNA and recombinant protein vaccines were reported to induce immuno-protection to live parasite challenge. Higher body weight gain, lower fecal oocyst shedding and reduced intestinal pathology were detected for immune protection. Jang et al. [32] reported that birds had lower oocyst concentration in droppings and reduced intestinal pathology after vaccination with Gam82 and challenged with *E. maxima* when compared with non-vaccinated and parasite-challenged groups. Xu et al. [33] determined that pcDNA3.0-TA4-IL-2 could decrease caecal lesions and body weight loss as well as produce an ACI of 192. Song et al. [34] reported that chickens immunised with pMP13 plasmid showed significantly lower number of oocysts following the challenge with *E. acervulina* compared to those in the negative controls. Similar results were detected in this study, both pVAX1-EmSAG and rEmSAG vaccines were successful in alleviating jejunal lesions, decreasing loss of body weight and the oocyst ratio.

The chick-anti-*Eimeria* specific antibodies have been previously documented to provide minor protection against coccidiosis. However, humoral immunity may also contribute to the formation of protective immune responses [35]. Furthermore, Wallach [36] pointed out that antibodies could inhibit parasite development and provide passive immune protection. Lin et al. [37] reported that birds immunised with the *E. tenella* rEF-1α protein exhibited higher specific antibodies concentration than the negative controls. In this research, the antibody titers of the EmSAG-immunised animals were higher than the negative controls. The findings of this investigation confirmed that EmSAG could induce humoral immune response.

IFN-γ is an important cytokine involved in the Th1-mediated immune response. Chicken IFN-γ could elicit lymphocytes and enhance expression of MHC class II antigens [38]. IFN-γ could also reduce sporozoites development without affecting the sporozoite invasion of host cells [39]. In previous research, higher titers of IFN-γ were detected in the EmMIC7 vaccinated birds [20]. In this study, higher IFN-γ titers in the vaccinated birds were also detected than those in the control birds. These results demonstrate that EmSAG could elicit Th1 cellular immune responses against *E. maxima*.

It has been noted that cell-mediated immunity is the most important immune response to *Eimeria* infection. In this study, the CD4^+^ and CD8^+^ percentages were higher in the groups immunised with pVAX1-EmSAG and rEmSAG protein, when compared to the control groups. This demonstrated that EmSAG might be able to stimulate cellular immunity.

IL-4 is known as a marker of the Th2 immune response [40] and has been reported as an important factor in protective immunity against parasite infections [41]. Tian et al. [42] reported that groups vaccinated with EmGAPDH exhibited higher concentrations of IL-4 compared to control groups injected with PBS and pVAX1 alone. The results of this study demonstrated an increased IL-4 level in the EmSAG-vaccinated birds compared to those in the negative control. Coupled with the high antibody concentration, these data indicate that EmSAG could stimulate humoral immune response to *E. maxima.*

A new class of T-helper cells known as Th17 cells is associated with interleukin IL-17 production [43]. In the avian immune system, IL-17 functions as a stimulator of cytokine productions [44]. It has been confirmed that co-vaccination of IL-17 with 3-1 E protein induced better protection against *E. acervulina* than 3-1 E alone [4]. Previously, it was reported that the immunisation of animals with DNA vaccines produced higher levels of IL-17 production [45]. However, IL-17 neutralised antibody treated birds showed enhanced IL-12 and IFN-γ expression [46]. In this research, a significant increment of IL-17 concentrations was detected ten days after the last immunisation. This finding coupled with the high IFN-γ titers, indicated that EmSAG could induce Th1 and Th17 response. However, the exact function of TH17 in immunisation against *Eimeria* spp. needs further investigation.

TGF-β is a cytokine that has been recognised as part of the immune suppression mechanism [47, 48]. TGF-β has been reported to induce protective immunity and increased TNF-α production [49, 50]. Hoan et al. [51] also reported that EbAMA1 could induce significantly higher concentrations of TGF-β1 and IL17 in the vaccinated groups. Likewise, in the current research, birds vaccinated with the rEmSAG protein and pVAX1-EmSAG showed higher concentrations of TGF-β1 than that of control groups. However, the exact function of TGF-β in protecting against coccidiosis needs further investigation.

Antibodies and cytokines have been shown to influence the protective immunity against coccidiosis infections. In previous reports, monoclonal antibodies showed the ability to reduce oocyst shedding and provide partial protection against *E. maxima* or *E. tenella* challenge infections [52, 53]. IL-4 could enhance the production of the antibody [54]. Chickens injected with recombinant IFN-γ showed improved protective immunity following *E. acervulina* infection [55–57]. Rose et al. [58] found that neutralising IFN-γ though monoclonal antibody could increase the output of oocysts and loss of body weight. Additionally, oocyst shedding was decreased in birds co-injected with IFN-γ or TGF-β with the 3-1E DNA vaccine compared to the birds inoculated with the DNA vaccine alone [59]. Lillehoj et al. [60] reported that co-vaccination with EtMIC2 and TGF-β significantly reduced oocyst shedding and enhanced weight gains beyond those injected by EtMIC2 alone. Zhang et al. [46] found that the IL-17 neutralised birds showed decreased fecal oocyst output and caecal lesion scores, as well as increased body weight gain. Geriletu et al. [44] reported that vaccination with IL-17A and MZP5-7 reduced oocyst shedding and decreased intestinal lesions following *E. tenella* challenge compared to inoculation with MZP5-7 alone. In this study, challenge experiments showed that the concentration of anti-EmSAG antibodies, IFN-γ, IL-4, TGF-β and IL-17 were increased in both the rEmSAG protein and pVAX1-SAG immunised groups. Additionally, the jejunal lesions, loss of body weight and oocyst production ratio were all decreased. These results indicate that the antibodies and cytokines played a role in the immune protection induced by the rEmSAG protein.

Localisation of the proteins is critical to understanding the role which they play in parasite binding and the invasion of the host cell [61, 62]. Previous studies reported that monoclonal antibodies were able to detect proteins on the parasite surface, such as EtSAG1 and the micronemes of the sporozoites and merozoites [63–65]. Jenkins et al. [66] showed the immune-mapped protein 1 could be detected in the sporozoites. Zhang et al. [31] found EaMIC3 on the apical tip of *E. acervulina* sporozoites. Our findings suggest that EmSAG is expressed in the sporozoite and merozoite stages of *E. maxima*, and might play an important role in the host invasion mechanism.

## Conclusions

In conclusion, our findings indicate that vaccination with EmSAG is capable of eliciting both humoral immunity and cell-mediated immunity, exploring a moderate protective immunity against *E. maxima*. This work suggests that EmSAG could be used as an effective vaccine candidate to resist *E. maxima* infection.

## Abbreviations

ACI: Anti-coccidial index
Cy3: Cyanine dyes 3
DAPI: 2-(4-amidinophenyl)-6-indolecarbamidine dihydrochloride
EmSAG: surface antigen gene of *Eimeria maxima*
IFN-γ: Interferon-γ
IgG-HRP: horseradish peroxidase labeled immunoglobulin G
IL-17: Interleukin-17
IL-4: Interleukin-4
IPTG: Isopropyl-B-D-thiogalactopyranoside
RT-PCR: Reverse transcription-polymerase polymerase chain reaction
SDS-PAGE: Sodium dodecyl sulfate polyacrylamide gel electrophoresis
TGF-β1: Transforming growth factor-β1
Th1: helper T cell 1
Th2: helper T cell 2
